# Fluid as a critical biomarker in neovascular age-related macular degeneration management: literature review and consensus recommendations

**DOI:** 10.1038/s41433-021-01487-0

**Published:** 2021-04-01

**Authors:** Laurent Kodjikian, Mariacristina Parravano, Andreas Clemens, Rosa Dolz-Marco, Frank G. Holz, Marion R. Munk, Massimo Nicolò, Federico Ricci, Rufino Silva, S. James Talks, Rohini Kumar Verma, Javier Zarranz-Ventura, Sandrine A. Zweifel

**Affiliations:** 1 Department of Ophthalmology, Croix-Rousse University Hospital, Hospices Civils de Lyon, Lyon, France; 2 UMR-CNRS 5510 Matéis, Villeurbanne, INSA de Lyon, Université Claude Bernard Lyon 1, University of Lyon, Lyon, France; 3 IRCCS - Fondazione Bietti, Rome, Italy; 4 Novartis Pharma AG, Basel, Switzerland; 5 Department of Cardiology and Angiology I, Heart Center Freiburg University, Faculty of Medicine, University of Freiburg, Freiburg im Breisgau, Germany; 6 Macula Unit, Oftalvist Clinic, Valencia, Spain; 7 Department of Ophthalmology, University of Bonn, Bonn, Germany; 8 Department of Ophthalmology, Inselspital, University Hospital Bern, Bern, Switzerland; 9 University Eye Clinic of Genoa DINOGMI, Ospedale Policlinico San Martino - IRCCS, Genoa, Italy; 10 Department of Experimental Medicine, University Tor Vergata, Rome, Italy; 11 Coimbra Institute for Clinical and Biomedical Research, Faculty of Medicine, University of Coimbra (ICBR-FMUC), Coimbra, Portugal; 12 Department of Ophthalmology, Centro Hospitalar e Universitário de Coimbra (CHUC), Coimbra, Portugal; 13 Association of Innovation and Biomedical Research in Light and Image (AIBILI), Coimbra, Portugal; 14 Newcastle upon Tyne Hospitals NHS Foundation Trust, Newcastle upon Tyne, UK; 15 Hospital Clínic de Barcelona, Barcelona, Spain; 16 Institut de Investigacions Biomediques August Pi i Sunyer (IDIBAPS), Barcelona, Spain; 17 Department of Ophthalmology, University Hospital Zurich, Zurich, Switzerland; 18 University of Zurich, Zurich, Switzerland

**Keywords:** Drug therapy, Prognostic markers, Eye manifestations

## Abstract

Current guidelines on the management of patients with neovascular age-related macular degeneration (nAMD) lack clear recommendations on the interpretation of fluid as seen on optical coherence tomography (OCT) imaging and the incorporation of this information into an ongoing disease treatment strategy. Our objective was to review current guidelines and scientific evidence on the role of fluid as a biomarker in the management of nAMD, and develop a clinically oriented, practical algorithm for diagnosis and management based on a consensus of expert European retinal specialists. PubMed was searched for articles published since 2006 relating to the role of fluid in nAMD. A total of 654 publications were screened for relevance and 66 publications were included for review. Of these, 14 were treatment guidelines, consensus statements and systematic reviews or meta-analyses, in which OCT was consistently recommended as an important tool in the initial diagnosis and ongoing management of nAMD. However, few guidelines distinguished between types of fluid when providing recommendations. A total of 52 publications reported primary evidence from clinical trials, studies, and chart reviews. Observations from these were sometimes inconsistent, but trends were observed with regard to features reported as being predictive of visual outcomes. Based on these findings, diagnostic recommendations and a treatment algorithm based on a treat-and-extend (T&E) regimen were developed. These provide guidance on the diagnosis of nAMD as well as a simple treatment pathway based on the T&E regimen, with treatment decisions made according to the observations of fluid as a critical biomarker for disease activity.

## 摘要

**积液作为新生血管性年龄相关黄斑变性治疗的关键性生物标志物: 文献回顾及共识建议**

摘要

目前关于新生血管性年龄相关性黄斑变性 (nAMD) 患者的管理指南中缺乏相干光断层扫描 (OCT) 中积液的阐释, 也没有明确的建议将该信息纳入正在制定的疾病治疗策略中。本文旨在总结目前积液作为生物标志物在nAMD中治疗作用的相关指南及科学证据, 并基于欧洲视网膜专家共识, 开发以临床需求为导向的具有实用价值的诊断和管理的算法。我们检索了PubMed数据库并查找自2006年以来发表的关于积液在nAMD中作用的文献。共筛选654篇文章, 纳入66篇文章以综述回顾。其中, 14项为治疗指南、共识陈述、系统综述及meta分析, 其中OCT被一致推荐作为nAMD的初始诊断和后续管理的重要工具。然而, 在提供建议时, 很少有指南会区分不同类型的液体。共有52篇文献报道了来自临床试验、研究和图表评论的主要证据。这些观察结果有时是不一致的, 但在预测视力方面有一定特征性的趋势。基于这些发现, 我们提出了诊断建议和基于T&E方案的治疗算法。这为nAMD的诊断提供了指导, 同时也为T&E方案提供了一个简单的治疗途径, 即根据观察到的积液作为疾病活动的关键生物标志物来决定治疗方案。

## Introduction

Since the widespread introduction of optical coherence tomography (OCT) for the visualisation of the back of the eye in patients with eye diseases such as neovascular age-related macular degeneration (nAMD), the evaluation of lesion morphology using OCT has become a key part of the clinical decision-making pathway [1]. Markers for disease activity based on OCT, including intraretinal and subretinal as well as subretinal pigment epithelium (RPE) fluid, are crucial for guiding management and treatment frequency of nAMD patients.

Recent advances in OCT technology have led to increases in speed and resolution that permit the detection of small structural changes to the retinal layers [2]. However, the interpretation of OCT images can be complex and challenging. Although this is an area of considerable scientific interest and extensive literature exists which attempts to evaluate the influence of different types of fluid on outcomes in nAMD, current guidelines may be lacking or open to misinterpretation when it comes to translating the diagnostic findings from an OCT into an ongoing disease treatment strategy. Clear treatment recommendations that consider both clinical and real-world considerations are therefore required.

The objective of this consensus article is to review the current guidelines and scientific evidence on the role of fluid as a biomarker in the management of nAMD and provide clinically useful recommendations based on a consensus of expert European retinal specialists. Furthermore, limitations of current literature and areas of further research are also highlighted.

## Methods

A preliminary review of the literature on the role of fluid in the management of nAMD was performed by Novartis in preparation for a roundtable discussion with European retinal specialists (consensus panel, consisting of LK, MP, RDM, FGH, MRM, MN, FR, RS, SJT, JZV and SAZ), held in Zurich, Switzerland (19 July 2019). During this initial meeting, the available scientific evidence—and the lack of it—were discussed, resulting in the proposal from the consensus panel to develop simplified treatment recommendations in nAMD. The literature review was subsequently repeated with revised search parameters and the updated results were subject to further review by the consensus panel during the development of the treatment recommendations, ensuring scientific rigour and unbiased interpretation. Novartis was not involved in the interpretation of the literature search results or the development of the treatment recommendations.

The repeated literature search of PubMed was performed according to the predefined search parameters shown in Table 1, with other relevant publications included from information sources such as recent congress presentations and educational resources. The resulting publications were screened by title and abstract for relevance and according to the following exclusion criteria: case reports and studies with fewer than 50 patients; opinion pieces other than expert consensus recommendations and guidelines; non-English language publications; and publication date prior to 2006. The scientific evidence that was retrieved by the search was tabulated and graded according to recent European guidance [3].

**Table 1:** Search parameters.

Parameters
(“age-related macular degeneration”[All Fields] OR AMD[All Fields]) AND fluid[All Fields]
AND date limits: 2006–2019
AND one or more of the secondary search terms:
• Visual acuity
• Visual function
• Vision
• Association *or* correlation *or* predictor *or* biomarker *and* visual acuity *or* visual function *or* vision
• Association *or* correlation *or* predictor *or* biomarker *and* disease progression
• Fluctuations *or* fluctuating *or* variability *and* CST *or* CRT *or* thickness
• Pathophysiology
• Diagnosis
• Management
• Anatomical
• Structural
• Prognosis

*AMD* age-related macular degeneration, *CRT* central retinal thickness, *CST* central subfield thickness.

The evidence was discussed by the consensus panel and used, along with their expert opinion and experience, to inform the development of a consensus management algorithm for patients with nAMD based primarily on observations of fluid from OCT monitoring. The nAMD-specific terminology used within this article follows recent consensus nomenclature for reporting nAMD data [4]. The term intraretinal fluid (IRF) is used throughout the document to standardise the different terms used to describe the presence of fluid within the retina including intraretinal cystoid oedema, intraretinal cysts, cystoid oedema, cystoid macular oedema and retinal fluid.

## Results

**Fig. 1:**
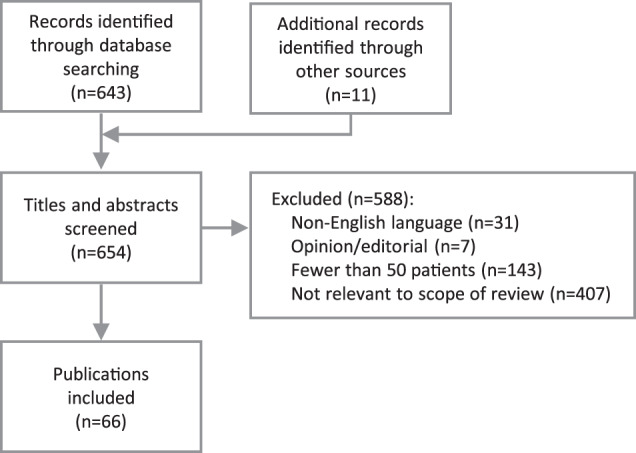
Literature review flow diagram. Sixty-six eligible publications were selected for inclusion.

The literature review was performed on September 25, 2019 (Fig. 1). After screening of 654 publications and excluding those that were not relevant or were outside the scope of the review, a total of 66 publications were included. Of these, 14 publications were treatment guidelines, consensus statements and systematic reviews or meta-analyses, while 52 publications reported primary evidence from clinical trials, studies, and chart reviews.

### Treatment guidelines

Six treatment guidelines from institutions in Europe and the USA were retrieved by the search. In the Royal College of Ophthalmologists’ (RCOphth) guidance on the use of ranibizumab in nAMD from 2009, new subretinal fluid (SRF) with or without haemorrhage is included as one criteria for treatment initiation, while their definition of disease activity for continuation of treatment includes IRF, SRF, sub-RPE fluid and haemorrhage [5]. In later guidelines on AMD from 2013, the RCOphth provided similar recommendations relating to fluid [6]. These latter guidelines have since been archived following the publication of the National Institute for Health and Care Excellence (NICE) guidelines on age-related macular degeneration (AMD) in January 2018. In these, NICE states that OCT should be offered to individuals with suspected active nAMD, or for ongoing monitoring of patients with active nAMD. No specific guidance is given with regard to fluid and treatment or management of the condition [7].

Few guidelines distinguished between types of fluid when providing recommendations, with the same retreatment approach generally recommended regardless of the type and location of fluid observed. One of the few that made a distinction between fluid types was the 2014 EURETINA guideline on nAMD, which advised that IRF, SRF and RPE detachments are important signs of neovascular activity independent of central retinal thickness (CRT), and that a ‘zero tolerance’ approach to OCT criteria is justified given the rapid progression of exudative features and progressive loss of vision when initiation of treatment is delayed in nAMD. However, longstanding persistent IRF should be considered a sign of irreversible retinal damage which should not prompt continued retreatment. Performing OCT was recommended as the most useful tool for evaluating morphological changes since it provides the most accurate reflection of the recurrence of disease activity. Qualitative morphology-based OCT data were considered to be more sensitive than current quantitative measurements such as CRT for detecting choroidal neovascularisation (now termed macular neovascularisation [MNV]) activity [8].

In the American Academy of Ophthalmology preferred practice pattern for AMD from 2015, there is no specific mention of how to interpret retinal fluid in diagnosis or follow-up, other than a statement that as-needed treatment should be based on the presence or absence of SRF or IRF [9]. Finally, recommendations on outcome measures for macular degeneration provided by the International Consortium for Health Outcomes Measurement and a group of experts in 2016 advised that the presence of IRF, SRF or haemorrhage attributable to neovascular lesion activity (as determined by the treating ophthalmologist) should be assessed at each clinic visit [10].

### Consensus statements

A number of expert consensus statements have provided guidance on the management of nAMD including recommendations relating to fluid and other anatomical parameters visualised using OCT. These are broadly consistent but differ in the detail of interpreting the various morphological features.

In 2011, a group of 22 European experts provided consensus recommendations for anti-vascular endothelial growth factor (VEGF) management of nAMD based on morphological criteria. Suggested retreatment criteria under a pro-re-nata (PRN) regimen included IRF, SRF, diffuse foveal thickening and expanding serous pigment epithelium detachment (PED). Criteria for delaying treatment included the absence of the above criteria, stable serous PED and stable IRF that has not responded to three intravitreal injections [11]. Notably, at the time of these recommendations, retreatment criteria were based on the assessment of a single transfoveal OCT image [11]. A committee of UK-based retinal experts published a consensus paper defining response to anti-VEGF therapy in nAMD in 2015. They noted that there is often little correlation between morphological and functional responses to anti-VEGF treatments, and so recommended a combination of morphology and function as the means of determining treatment response, with the morphology component defined as IRF, SRF and retinal thickening [12].

In a 2017 expert round-table consensus on the treatment of nAMD with aflibercept in the second year of therapy, fluid was a recommended consideration when making the decision to maintain a fixed regimen or move to a treat-and-extend (T&E) dosing schedule. The criteria for not extending the treatment interval included persistent macular fluid with stable vision, recurrent fluid, and decrease in vision in the presence of fluid. Extension of intervals between treatments was recommended for eyes with no macular fluid and stable vision [13].

A recent Greek consensus statement on the management of nAMD recognised the importance of morphological signs of disease activity observed using OCT, which the authors note correspond to early signs of recurrence prior to measurable loss of VA. The main anatomic parameters to be taken into consideration according to their recommendations were CRT, SRF, IRF, anatomy of the outer retinal layers and PED [14].

### Systematic reviews

Four systematic reviews were included in the literature review. The earliest of these was a systematic review on OCT for diagnosis, monitoring and guiding treatment for nAMD by Mowatt and colleagues from 2014, which concluded that strategies involving OCT alone for diagnosis and/or monitoring were unlikely to be cost-effective, while those that also included fluorescein angiography (FA) and other imaging techniques were more likely to be considered cost-effective. However, many of the studies included in this review used older, time-domain OCT technology which may have compromised the specificity of the technique in terms of detecting active nAMD. For the purposes of this review, nAMD was considered to be active or inactive, with no specific discussion relating to fluid [15]. In contrast, a review by Schmid-Erfurth and Waldstein from 2016 provided detailed information on imaging biomarkers in nAMD. The authors concluded that CRT is an inferior prognostic biomarker for guiding retreatment compared with localisation of fluid in different compartments, including IRF and SRF. IRF at baseline is negatively associated with VA, while SRF at baseline (i.e., in naive patients) is associated with superior visual benefits and a lower rate of progression towards atrophy. The finding of SRF is associated with all lesion types and is typically the first exudative sign in Type 1 lesions. RPE detachment was identified as unresponsive to therapy and responsible for visual decline [16]. A later systematic review on OCT in the management of AMD by the same group provided a detailed discussion of morphological features indicative of disease activity, but was accompanied by no clear guidance for treatment [17].

A systematic review of the evidence on using morphological predictors to modify treatment protocols in nAMD was performed by Ashraf et al. [18], finding that a good response in terms of reduction in SRF at 12 weeks predicted good visual outcomes, but that patients with PED and IRF achieved smaller visual gains and their treatment intervals should be extended with caution.

### Primary evidence

The 52 primary publications of clinical trials and studies retrieved by the literature search were reviewed for relevant detail on the role or impact of fluid in nAMD. Of these, one publication was the primary output of a randomised controlled trial (RCT) [19], 21 publications were post-hoc analyses, exploratory analyses and prospective cohort studies related to several medium and large RCTs (ABC trial [20], PIER [21], CATT [22–27], MONT BLANC [28], EXCITE [29, 30], GEFAL [31], VIEW 1 and 2 [32–36] and HARBOR [37–39]), 5 publications described prospective, non-randomised studies, and the remaining 25 publications were retrospective chart reviews and case series. Table 2 provides a summary of the studies and their findings, while Table 3 compares features of several of the key RCTs of anti-VEGF therapy in nAMD, including the retreatment criteria applied to the flexible treatment arms or phases of these trials.

**Table 2:** Overview of clinical trials and studies investigating fluid in nAMD.

Reference	Study design	Patient population	Number of patients	Number of eyes	Key findings	Level of evidence
Keane et al. [57]	Retrospective, cross-sectional study	Newly diagnosed with nAMD	216	216	• Increased total volume of SRT correlated with decreased VA (*P* < 0.0001) • Increased thickness of the neurosensory retina at the foveal centre point modestly correlated with decreased VA (*P* = 0.0004) • No significant association detected between VA and total volume of SRF or PED	4
Dadgostar et al. [77]	Retrospective, interventional case series	Treatment-naive nAMD undergoing ranibizumab monotherapy	124	131	• Resolution of IRF and SRF did not correlate with the degree of vision improvement upon treatment with ranibizumab	4
Kashani et al. [71]	Retrospective, interventional case series	Treatment-naive nAMD beginning treatment with ranibizumab	53	53	• Increased outer nuclear layer volume associated with decreased VA (*P* = 0.002) • Increased SRT thickness significantly correlated with decreased VA (*P* = 0.001)	4
Unver et al. [78]	Retrospective, interventional case series	Treated with ≥3 injections of bevacizumab and followed for ≥6 months	48	50	• Global macular acuity associated with change in SRF thickness on OCT, but SRF thickness alone was not sufficient to predict outcomes	4
Keane et al. [20] (ABC trial)	Cross-sectional study of patients in an RCT	Newly diagnosed nAMD	122	122	• Increased SRT volume correlated with decreased contrast sensitivity (*P* = 0.001) • SRF volume modestly correlated with contrast sensitivity (*P* = 0.004) • Increased retinal thickness at the foveal centre correlated with decreased VA (*P* < 0.001)	4
Mariani et al. [73]	Consecutive case series	Treatment-naive nAMD beginning treatment with ranibizumab	99	ns	• Loss of VA after the initial 3 months was associated with the presence of PED at baseline (*P* = 0.01)	4
Silva et al. [79]	Single-centre, prospective, observational, longitudinal 2-year study with 1-year extension	nAMD in the non-study eye and early age-related maculopathy in the fellow (study) eye	62	62	• OCT findings did not show any correlation with an increased risk of conversion to nAMD	4
Kolb et al. [55]	Retrospective, interventional case series	Treated with anti-VEGF and followed for ≥12 months	ns	75	• CRT, integrity of the ellipsoid zone or presence of IRF or SRF was not predictive of VA outcome after 12 months of treatment • Eyes with thicker CRT at baseline had a greater reduction in CRT which was associated with better VA outcomes	4
Padnick-Silver et al. [53]	Prospective, observational, non-randomised study	nAMD in the non-study eye and non-exudative macular degeneration in the fellow (study) eye	79	79	• In 13/15 patients who developed nAMD in the study eye over 2 years, disease progression was identified on OCT before FA or examination showed changes • New or growing sub-RPE fluid and small areas of SRF or IRF were early signs of conversion to nAMD	4
Wickremasinghe et al. [50]	Prospective, consecutive, non-randomised study	Treatment-naive subfoveal MNV secondary to AMD treated with ranibizumab and/or bevacizumab	ns	214	• The location of fluid at baseline did not significantly influence the likelihood of BCVA improvement at 12 months • Eyes with RPE hyperreflectivity at baseline had a greater likelihood of BCVA loss (*P* = 0.006) and poorer final BCVA (*P* = 0.02) than those without • Eyes with residual IRF at 3 months had worse BCVA at 12 months than those with no fluid or with SRF alone	4
Brown et al. [21] (PIER)	Post-hoc analysis of data from an RCT	Primary or recurrent subfoveal MNV secondary to AMD who had not received anti-VEGF therapy for ≥1 month	87	87	• Absence of fluid on OCT at months 5 and 8 was predictive of greater BCVA gains at month 24 (*P* ≤ 0.045)	4
Jaffe et al. [22] (CATT)	Prospective cohort study within an RCT	Treatment-naive active MNV due to AMD treated with ranibizumab or bevacizumab	1142	1142	• At all time points, eyes with residual IRF (especially foveal) had worse mean VA than those without IRF (*P* < 0.0001) • Eyes with abnormally thin (<120 µm) or thick (>212 µm) retinas had worse VA than those with normal thickness retinas • Monthly treatment eliminated fluid of any type during the first year more effectively than PRN treatment (*P* = 0.002)	4
Shin et al. [47]	Retrospective, consecutive case series	Patients treated with anti-VEGF	267	ns	• In patients refractory to anti-VEGF treatment, a subgroup with extensive IRF had limited visual improvement and poor final VA	4
Ritter et al. [28] (MONT BLANC)	Analysis of data from RCT comparing ranibizumab versus ranibizumab plus PDT	Treatment-naive AMD-related active subfoveal MNV classified as Type 2, mixed Type 1 and Type 2 or Type 1	255	255	• CRT did not consistently correlate with BCVA, particularly during the maintenance phase • IRF at baseline had the strongest negative predictive value for BCVA gain in both study groups (*P* = 0.006) • SRF at baseline was predictive of a higher number of ranibizumab injections (*P* < 0.01) • PED at baseline was associated with a higher number of ranibizumab injections in the monotherapy group (*P* < 0.01)	2
Simader et al. [29] (EXCITE)	Subanalysis of data from RCT comparing different doses and regimens of ranibizumab	Treatment-naive subfoveal MNV	353	ns	• IRF at baseline was associated with lower BCVA that remained lower over the study period • Recurrence of SRF during follow-up showed a trend for a negative effect on visual function (*P* = 0.06) • PED at baseline was predictive of a poor visual outcome only in combination with IRF and SRF	2
Ying et al. [23] (CATT)	Cohort study within an RCT	Treatment-naive active choroidal neovascularisation due to AMD treated with ranibizumab or bevacizumab	1030	1030	• Patients with sustained VA loss at 2 years had higher proportions of IRF (*P* < 0.001), subretinal HRM (*P* < 0.001), retinal thinning (*P* < 0.001), and retinal thickening (*P* < 0.001) than those without sustained VA loss • The presence of IRF not at the foveal centre at baseline was associated with an increased risk of sustained VA loss	4
Gianniou et al. [80]	Retrospective, consecutive chart review	Treatment-refractory nAMD, (persistent IRF or SRF despite monthly ranibizumab injections for ≥12 months)	74	76	• Maintained VA gains were possible even with refractory fluid • Refractory IRF was associated with poorer anatomical and functional outcome than SRF	4
Regillo et al. [37] (HARBOR)	Retrospective, exploratory analysis of RCT data	Treatment-naive active subfoveal nAMD treated with ranibizumab monthly or PRN	500	500	• Presence of SRF at baseline was associated with an increased likelihood of 20/40 or better vision at month 12 of treatment • Smaller total MNV leakage area with SRF present at baseline predicted a greater likelihood of BCVA gain >15 letters at month 12 • A thicker retina at baseline (>118.25 µm) was associated with greater injection requirements over 12 months	4
Schmidt-Erfurth et al. [32] (VIEW 1 and 2)	Post-hoc analysis of data from an RCT	Treatment-naive nAMD treated with ranibizumab or aflibercept	1240	ns	• The sub-RPE lesion underlying PED appears to be the primary indicator for progressive disease activity • Secondary cystoid degeneration is the most relevant imaging marker for visual function	4
Shin et al. [56]	Retrospective, consecutive chart review	80 typical nAMD and 61 PCV treated with ranibizumab, plus 121 controls	204	262	• In patients with typical nAMD, those classified as having a thin choroid (<177 µm) had a higher prevalence of IRF/SRF and less visual gain at 12 months than those with a medium choroid (177–340 µm) (thin vs medium choroid, p < 0.0001)	4
Arnold et al. [43] Guymer et al. [19] (FLUID)	RCT	Treatment-naive active subfoveal MNV secondary to nAMD treated with ranibizumab	349	349	• Study participants treated with a protocol that tolerates a degree of SRF achieved non-inferior BCVA to those treated with the aim of resolving all SRF completely	1
Casalino et al. [40]	Retrospective analysis	nAMD treated with ranibizumab or aflibercept and with ≥12 months of follow-up	117	121	• ELM disruption at baseline and month 12 was a negative predictive factor for final BCVA (*P* = 0.001 and *P* < 0.001, respectively) • SRF at month 12 was a positive predictor for final BCVA (*P* = 0.007)	4
Chatziralli et al. [49]	Retrospective, consecutive case series	nAMD with insufficient response to ranibizumab switched to aflibercept	431	447	• Increasing CRT, presence of PED and presence of IRF were associated with a poor visual outcome following switching anti-VEGF agent • The presence of SRF only did not affect VA	4
Dervenis et al. [45]	Retrospective case series	Newly diagnosed nAMD treated with ranibizumab	62	62	• IRF at baseline was associated with worse VA outcomes in month 4 (*P* = 0.045) but not month 6 • PED did not affect treatment response	4
Jaffe et al. [33] (VIEW 1 and 2)	Post-hoc analysis of data from an RCT	Treatment-naive nAMD treated with ranibizumab or aflibercept	1815	1815	• In eyes with persistent IRF or SRF, BCVA gains were greater with monthly versus bimonthly dosing (*P* < 0.05)	4
Koizumi et al. [59]	Retrospective, consecutive, interventional case series	Treatment-naive nAMD treated with aflibercept	144	ns	• Decrease in subfoveal choroidal thickness was significantly associated with VA gain at 12 months in eyes with PCV (*P* = 0.0087) but not those with typical nAMD (*P* = 0.17)	4
Lee et al. [75]	Retrospective chart review	44 with PCV and 44 with nAMD treated with anti-VEGF for ≥6 months	88	ns	• VA after treatment was associated with number of subretinal HF (*P* = 0.046)	4
Moshfeghi et al. [34] (VIEW 1 and 2)	Post-hoc analysis of data from an RCT	Treatment-naive nAMD treated with ranibizumab or aflibercept	ns	1465	• BCVA change at week 52 was independent of retinal fluid status (presence or absence of SRF or IRF) at week 12	4
Segal et al. [52]	Retrospective chart review	Treatment-naive nAMD treated with bevacizumab	73	76	• Eyes with >20 HF, HF in the inner retinal layers, increased CRT and IRF had the worst BCVA at 12 months	4
Segal et al. [41]	Retrospective cohort study	Treatment-naive nAMD treated with 3 injections of bevacizumab and follow-up of ≥4 months	105	105	• SRF width was significantly positively correlated with improved BCVA following treatment (*P* = 0.018) • Eyes with IRF had poor visual outcomes	4
Shah et al. [24] (CATT)	Prospective cohort study within an RCT	Treatment-naive active choroidal neovascularisation due to AMD treated with ranibizumab or bevacizumab	1185	ns	• Eyes with CMO at baseline had worse VA at baseline and 2 years than those with IRF but no CMO or those with neither IRF or CMO, but gains in VA were similar between groups	4
Sharma et al. [25] (CATT)	Prospective cohort study within an RCT	Treatment-naive active choroidal neovascularisation due to AMD treated with ranibizumab or bevacizumab	1185	ns	• At 2 years, eyes with IRF in the foveal centre had worse mean VA than eyes without IRF (*P* = 0.0001) • At 2 years, eyes with retinal thickness <120 μm had worse VA compared with those with retinal thickness 120–212 μm and >212 μm (*P* < 0.0001)	4
Waldstein et al. [35] (VIEW 1 and 2)	Post-hoc analysis of data from an RCT	Treatment-naive nAMD treated with ranibizumab or aflibercept	1815	1815	• Presence of IRF was associated with lower VA at baseline and 2.77 letters less BCVA change from baseline at week 52	4
Waldstein et al. [30] (EXCITE)	Post-hoc analysis of data from RCT comparing different doses and regimens of ranibizumab	Treatment-naive subfoveal MNV	353	ns	• Baseline SRF was predictive of positive BCVA change at month 12 (*P* = 0.05) • Baseline IRF were predictive of negative BCVA change at month 12 (*P* = 0.05) • Patients without SRF at baseline had higher BCVA gains with frequent dosing versus infrequent dosing, but in those with SRF at baseline VA gains were similar with frequent or infrequent dosing	4
Wickremasinghe et al. [44]	Prospective, single-arm study	nAMD treated with ranibizumab via a treat-and-extend protocol with follow-up of ≥12 months	99	103	• At 12 months, IRF/SRF was present in 37.3% of cases when ≥5 letters BCVA was lost • New occurrences of IRF or SRF were more likely to lead to BCVA loss, compared with no or persistent fluid (*P* < 0.001) • Small amounts of persistent fluid could be tolerated without compromising vision	4
Lee et al. [46]	Retrospective analysis	Typical nAMD treated with ranibizumab	61	65	• At month 12, increased volume of IRF was associated with poor BCVA (*P* = 0.01) • Volume of IRF at baseline was a significant predictor of BCVA at month 12 (*P* = 0.01)	4
Sagiv et al. [48]	Retrospective case series	nAMD treated with ≥30 anti-VEGF injections	61	67	• Eyes with worse final VA had more IRF (*P* = 0.05)	4
Vogel et al. [42]	Retrospective, consecutive chart review	nAMD treated with anti-VEGF with follow-up of ≥6 months	ns	131	• At 6 and 12 months, visual improvement was associated with SRF (*P* = 0.02) Visual worsening was associated with retinal PED (*P* = 0.04) and IRF (*P* = 0.01)	4
Kodjikian et al. [31] (GEFAL)	Analysis of data from an RCT	Treatment-naive subfoveal AMD treated with ranibizumab or bevacizumab	404	404	• IRF at baseline and central subfield macula ≤277 µm at baseline were associated with a lower BCVA at 1 year and lower BCVA gains (all *P* ≤ 0.01)	2
Pokroy et al. [51]	Retrospective, consecutive chart review	Treatment-naive centre-involved nAMD treated with bevacizumab	73	73	• Baseline presence of IRF, presence of subretinal HRM, well-defined subretinal HRM borders, and thick subretinal HRM were all significantly predictive of poorer 12-month VA	4
Schmidt-Erfurth et al. [38] (HARBOR)	Post-hoc analysis of data from an RCT	Treatment-naive active subfoveal nAMD treated with ranibizumab monthly or PRN	614	614	• Horizontal extension of IRF in the foveal region baseline was the most relevant biomarker for baseline BCVA • Morphologic features were largely not predictive of BCVA outcome at month 12	4
Ying et al. [26] (CATT)	Secondary analysis of data from a cohort study within an RCT	Treatment-naive active choroidal neovascularisation due to AMD treated with ranibizumab or bevacizumab	647	ns	• Absence of baseline SRF was associated with worse VA (*P* = 0.03) and more VA loss (*P* = 0.03) at 5 years • Absence of RPE elevation was associated with higher likelihood of ≥3-line gain at 5 years	4
Fulcher et al. [54]	Retrospective analysis	Treatment-naive nAMD treated with aflibercept	69	72	• Change in CRT and the location of fluid at baseline were not useful factors to predict long-term outcome	4
Hu et al. [39] (HARBOR)	Post-hoc analysis of data from an RCT	Fellow eyes of patients with treatment-naive active subfoveal nAMD treated with ranibizumab monthly or PRN	1097	1097	• In the majority of 92 eyes with new-onset exudation, HF and PED were present 1 month before conversion • Volumes of IRF fluid, SRF, subretinal HRM and PED significantly increased at the onset of exudation	4
Jaffe et al. [27] (CATT)	Cohort study within an RCT	Treatment-naive active choroidal neovascularization due to AMD treated with ranibizumab or bevacizumab	523	ns	• At 5 years, subretinal HRM, thinner retina (both *P* < 0.001) and IRF (*P* < 0.05) were independently associated with worse VA	4
Khurana et al. [36] (VIEW 1 and 2)	Post-hoc analysis of data from an RCT	Treatment-naive nAMD treated with ranibizumab or aflibercept	1551	1551	• Absence of retinal fluid and leakage at week 52 were significantly associated (both *P* < 0.0001) with dosing intervals of ≥12 weeks	4
Kim et al. [72]	Retrospective analysis	Newly diagnosed with type 3 neovascularisation and treated with anti-VEGF therapy	ns	137	• In patients with abrupt visual loss of ≥5 lines, this was associated with development of or increase in the height of PED with fluid in 36.4% patients	4
Kumar et al. [60]	Retrospective, consecutive case series	Treatment-naive nAMD followed for 1 year and treated with anti-VEGF therapy	62	ns	• Increased SFCT at baseline statistically significantly correlated with a higher number of injections at 1 year (*P* = 0.004) • Eyes with SFCT >1 standard deviation above the mean required 50% more injections compared with others • There was no association between baseline SFCT and VA at 1 year (*p* = 0.2)	4
Lai et al. [74]	Retrospective case series	Treatment-naive nAMD treated with ranibizumab or aflibercept for ≥1 year	126	126	• BCVA improvement at 1 year was negatively associated with PED at baseline (*P* = 0.031) and with IRF (*P* < 0.001) or PED (*P* = 0.002) at month 12	4
Lin et al. [81]	Retrospective, consecutive, case–control study	Treatment-naive patients with nAMD who achieved ER while on a PRN anti-VEGF regimen (*n* = 77 eyes) or patients with nAMD who didn’t achieve ER (*n* = 84 eyes)	70	77	• ER was achieved earlier in eyes with isolated IRF (*P* = 0.045) at baseline • Thinner choroid at baseline increased the likelihood of achieving ER (*P* = 0.004)	3
Roh et al. [58]	Prospective, cross-sectional study	Diagnosis of AMD with no intraocular procedure in the previous 90 days (*n* = 102) or control (*n* = 46)	77	148	• Reduced retinal thickness was associated with decreased mean retinal sensitivity (*P* < 0.0001) • SRF within the 10° diameter circle of the macula was associated with decreased retinal sensitivity (*P* < 0.05)	3

Levels of evidence: 1, Randomised clinical trial with low study errors or a meta-analysis; 2, Randomised clinical trial with high study error, usually ‘underpowered’; 3, Clinical trial including a control group, with non-random treatment allocation; 4, Interventional case series; 5, Interventional case report [3].

*BCVA* best-corrected visual acuity, *CMO* cystoid macular oedema, *CRT* central retinal thickness, *ELM* external limiting membrane, *ER* extended remission, *IRF* intraretinal fluid, *FA* fluorescein angiography, *HRM* hyperreflective material, *HF* hyperreflective foci, *MNV* macular neovascularisation, *nAMD* neovascular age-related macular degeneration, *ns* not specified, *PCV* polypoidal choroidal vasculopathy, *PDT* photodynamic therapy, *PED* pigment epithelial detachment, *PRN* pro re nata, *RCT* randomised controlled trial, *SFCT* subfoveal choroidal thickness, *SRF* subretinal fluid, *SRT* subretinal tissue, *VA* visual acuity.

**Table 3:** Retreatment criteria in key randomised controlled trials of anti-VEGF therapies in nAMD.

Study	Regimen in flexible dosing arm	OCT modality	Retreatment criteria
CATT [82]	1 mandatory injection followed by PRN dosing	Time domain	Signs of active neovascularisation defined as fluid on OCT, new or persistent haemorrhage, decreased VA compared with previous examination, or dye leakage or increased lesion size on FA
HARBOR [83]	3 mandatory monthly injections followed by PRN dosing	Spectral domain	≥5-letter decrease in vision from the previous visit or any evidence of disease activity on OCT (e.g., IRF, SRF, or sub-RPE fluid)
GEFAL [84]	3 mandatory monthly injections followed by PRN dosing	Spectral domain or time domain	At least one of: loss of ≥5 letters from the previous visit with no obvious atrophy or subretinal fibrosis and with fluid on OCT; and/or active exudation on OCT (SRF unless stable since the last 3 monthly injections, macular oedema with IRF, or increase in central subfield macular thickness of at least 50 µm compared with the previous examination); and/or increased MNV area or persistence of leakage on angiography since the previous visit; and/or new or persistent subretinal or intraretinal macular haemorrhage
VIEW 1 and 2 [36]	PRN dosing from week 52 to week 96	Time domain	At least one of: new or persistent fluid on OCT, increase in central subfield thickness ≥100 µm compared with the lowest previous value, loss of ≥5 ETDRS letters from the best previous score in conjunction with recurrent fluid on OCT, new-onset classic neovascularisation, new or persistent leak on FA, new macular haemorrhage, or time lapse of at least 12 weeks since the previous injection
FLUID [19]	3 mandatory monthly injections followed by a T&E regimen	Spectral domain	Loss of BCVA of ≥5 letters from the best BCVA recorded since baseline, new retinal haemorrhage, or presence of fluid on OCT. For the intensive arm, fluid was defined as the presence of any IRF, SRF, or both. For the relaxed arm, fluid was defined as the presence of any IRF and any SRF of >200 µm in height at the subfoveal centre

*anti-VEGF* anti-vascular endothelial growth factor, *BCVA* best-corrected visual acuity, *ETDRS* Early Treatment Diabetic Retinopathy Study, *IRF* intraretinal fluid, *FA* fluorescein angiography, *MNV* macular neovascularisation, *nAMD* neovascular age-related macular degeneration, *OCT* optical coherence tomography, *PRN* pro re nata, *RPE* retinal pigment epithelium, *SRF* subretinal fluid, *T&E* treat-and-extend, *VA* visual acuity.

Several studies reported that the presence of baseline SRF predicts a good response to anti-VEGF treatment, resulting in favourable visual outcomes [25, 26, 37, 40–42]. Evidence also suggests that small amounts (defined by the FLUID study as less than 200 µm) of residual stable SRF can be tolerated without impact on VA [19, 43]. However, one study reported that recurrent SRF is predictive of a poor functional prognosis [44]. A substantial number of studies reported consistent findings indicating that the presence of IRF (at baseline or recurring) is predictive of a poor prognosis [22, 23, 25, 27, 31, 41, 42, 44–52]. New or growing sub-RPE fluid is reported in one study as being an early sign of conversion to nAMD [53], while another study found an increase in sub-RPE fluid to be a marker for progressive disease activity which warrants treatment [32]. While this could be considered a useful predictive biomarker if observed over time, it should be noted that the presence of sub-RPE fluid in a single OCT scan without the presence of SRF and IRF is not necessarily indicative of disease progression.

Several publications found the type or spatial localisation of fluid to have limited prognostic value in terms of predicting response to anti-VEGF therapy [33, 38, 54, 55]. In the VIEW studies, a post-hoc analysis reported that BCVA change from baseline to week 52 was independent of the presence or absence of fluid at week 12 [34]. However, contrasting evidence from the PIER study suggests that an absence of fluid on OCT is predictive of greater BCVA gains with anti-VEGF treatment [21].

A significant number of studies found either abnormally thick or abnormally thin retinas to be associated with poor outcomes [20, 22, 25, 27, 31, 49, 52, 56, 57]. Reduced retinal thickness has been associated with decreased retinal sensitivity [58], and an increased total volume of subretinal tissue has been correlated with decreased VA or contrast sensitivity [20, 57]. In a retrospective study of patients initially treated with ranibizumab and then switched to aflibercept, subfoveal thickening and increased retinal central subfield thickness were reported to be predictive of poor prognosis in non-treatment naive patients [49]. In contrast, however, two studies reported that change in retinal thickness is not predictive of treatment outcomes [54, 59].

Finally, a small number of publications commented on correlations between fluid and required anti-VEGF injection frequency. Two publications reported that a thicker retina at baseline was associated with greater injection requirements [37, 60], while another stated that the presence of SRF was predictive of the need for a higher injection frequency [28]. A post-hoc analysis of the VIEW studies reported that the absence of retinal fluid at 1 year was predictive of the ability to achieve extended treatment intervals of at least 12 weeks [36].

### Algorithm for the management of nAMD

**Fig. 2:**
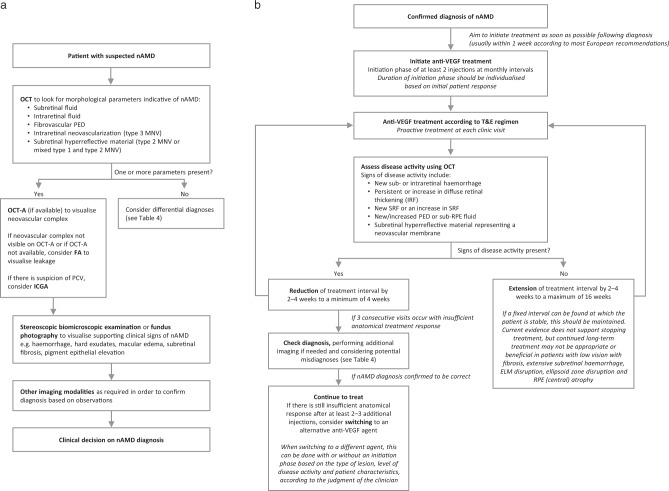
Algorithm for the management of nAMD: recommendations by the consensus panel. **a** Diagnosis. **b** Management according to a treat-and-extend regimen. anti-VEGF anti-vascular endothelial growth factor, ELM external limiting membrane, ICGA indocyanine green angiography, IRF intraretinal fluid, FA fluorescein angiography, MNV macular neovascularisation, nAMD neovascular age-related macular degeneration, OCT optical coherence tomography, OCT-A optical coherence tomography angiography, PCV polypoidal choroidal vasculopathy, PED pigment epithelial detachment, RPE retinal pigment epithelium, SRF subretinal fluid, T&E treat and extend.

Based on the available scientific evidence described above and the experience of the consensus panel, an algorithm for the most optimal management of patients with nAMD based on fluid observed using OCT and other imaging technologies is recommended, irrespective of country guidance and resource constraints, as shown in Fig. 2.

### Diagnosis and diagnostic techniques

The consensus panel agreed that morphological parameters observed on OCT are the most important criteria in routine clinical practice for the diagnosis of nAMD. The whole stack of images should be used, to give as full a picture as possible. The characteristic features considered to be indicative of nAMD are SRF, IRF and fibrovascular PED **(**Fig. 2a). OCT can permit differentiation of the type of MNV and location of fluid, but at baseline many cases also require additional information from other imaging modalities in order to confirm the diagnosis. If available, OCT-angiography (OCT-A) is considered to be valuable in order to visualise the neovascular complex. If OCT-A is not available, FA can be used to visualise leakage from the lesion, but is otherwise no longer judged to be a mandatory technique for nAMD diagnosis in all AMD cases. However, clinical signs visualised using biomicroscopy or fundus photography/examination are still considered useful to support the diagnosis. In cases where PCV or type 3 MNV is suspected, ICGA and OCT-A are recommended to confirm this diagnosis. In addition to the morphological and clinical signs of nAMD, patient age over 50 years is an important criterion for a diagnosis of nAMD.

A range of conditions have the potential to masquerade as nAMD. Table 4 lists a number of these potential misdiagnoses or pitfalls. Of these, the most common are adult-onset foveomacular vitelliform dystrophy and central serous chorioretinopathy. When examining a patient with putative nAMD, the clinician should be aware of and exclude these common differential diagnoses.

**Table 4:** Potential misdiagnoses for nAMD.

Category	Condition
Inherited retinal diseases	Pattern dystrophy with pigment epithelial detachment
	Adult-onset foveomacular vitelliform dystrophy
	Macular telangiectasia type 1
	MNV secondary to Sorsby fundus dystrophy
	MNV secondary to Stargardt disease
Trauma and infection	MNV secondary to presumed ocular histoplasmosis syndrome
	MNV secondary to choroidal rupture
	MNV secondary to trauma/laser pointers
Chorioretinal uveitis syndromes	Inflammatory MNV
	Chorioretinitis
	MNV secondary to punctate inner choroidopathy
Retinopathies	Diabetic maculopathy
	Central serous chorioretinopathy
	Pachychoroid neovasculopathy
	Myopic MNV
	Retinal vein occlusion
	Non-neovascular AMD, e.g., avascular PEDs with or without pockets of SRF
Neurodegenerative conditions	Macular telangiectasia type 2
Other	Perifoveal exudative vascular anomalous complex
	Macroaneurysm
	Epiretinal membrane
	Vitreomacular traction
	MNV secondary to previous laser photocoagulation in cases with concomitant DMO
	MNV secondary to angioid streaks
Degenerative structural features that mimic neovascular activity	Outer retinal tubulation
(non-specific for AMD)	Plateau sign
	Apoptotic cysts
	Pseudocysts
	Hyporeflective wedge
	Draping of drusen

*AMD* age-related macular degeneration, *DME* diabetic macular oedema, *MNV* macular neovascularization, *nAMD* neovascular age-related macular degeneration, *PED* pigment epithelial detachment, *SRF* subretinal fluid.

### Treatment

The agreement of the consensus panel was that, regardless of the anti-VEGF agent used, T&E is the recommended regimen for the management of nAMD because it provides comparable clinical outcomes to fixed monthly or bimonthly injections with a reduction in injection burden compared with fixed dosing [61, 62]. T&E also provides a reduction in the number of clinic visits compared with PRN with monthly monitoring, provided that a one-step visit (with follow-up and injection on the same day) is possible. The potential for development of atrophy with intensive anti-VEGF therapy was considered to be less of a concern than the likelihood of visual acuity loss resulting from undertreatment, since a link between the number of injections and the risk of developing atrophy has never been proven. On the contrary, current evidence suggests that anti-VEGF therapy is not a significant risk factor for the development of macular atrophy. For example, a post-hoc analysis of data from the HARBOR study reported no association of number of ranibizumab injections with macular atrophy development, and no significant association between regimen (monthly vs PRN treatment) and macular atrophy development [63]. Another post-hoc analysis of data from the same trial using Classification of Atrophy Meetings (CAM) group atrophy criteria found no differences in the incidence or progression rates of new macular atrophy among study arms, anti-VEGF doses, or treatment regimens [64]. However, there is evidence that neovascularisation type may be associated with the development of atrophy, with patients with type 1 MNV at baseline less likely to develop atrophy than eyes with other forms of MNV [65]. In contrast, patients with type 3 MNV and subretinal drusenoid deposits at baseline have a high risk of atrophy development [66].

Treatment with anti-VEGF therapy should be initiated as soon as possible once the diagnosis of nAMD is made. Guidelines and institutional guidance vary throughout Europe, with most recommendations advising that treatment should take place within 1 week of referral. The NICE guidelines mandate treatment within 14 days of referral, but specify that referral should take place within 1 working day of diagnosis [7]. Treatment should begin with an initiation phase before the clinician considers extending the treatment interval (Fig. 2b). This usually consists of three injections given at monthly intervals, but in some circumstances (as seen in real-world datasets [67]) could entail just two injections depending on the response of the individual patient. After that, the patient is evaluated for extension criteria, and the treatment interval can be increased by 2–4 weeks at a time.

Treatment should be given proactively at each visit—a key aspect of T&E treatment design. The decision on whether the treatment interval should be extended, retained, or reduced is also made at each visit, and is based on disease activity as assessed using OCT. The signs of disease activity that should trigger a reduction in treatment interval include new haemorrhage beneath or within the retina, new or persistent IRF, new or increased SRF, increased size of PED, or the presence of subretinal hyperreflective material which would indicate the presence of a neovascular membrane. If one or more of these signs are present, the treatment interval should be reduced by 2–4 weeks, to a minimum of 4 weeks. However, in a minority of cases with recurrent disease activity, the clinician may feel that an extension or reduction of 1 week might be more appropriate.

If disease activity is observed at three consecutive visits, with no sign of anatomical and/or functional improvement, the clinician should consider whether the initial diagnosis of nAMD was correct, using additional imaging modalities to provide more information if necessary. It might be possible that the patient is not a non-responder but has instead been misdiagnosed for nAMD. If further investigation confirms the original diagnosis, then the consensus panel recommends that treatment should be continued for at least 2 to 3 additional injections at the minimum interval permitted by the product label before a switch to an alternative anti-VEGF therapy is considered. If a patient is switched to a different anti-VEGF therapy due to lack of efficacy, this should be done with a new initiation phase. However, a simulated switching study has suggested that continuation of initial therapy will, in many cases, result in a gradual improvement or stabilisation similar to that commonly reported following a therapy switch in published anti-VEGF switching studies [68].

If there is no evidence of disease activity at the treatment visit, the clinician may consider extending the treatment interval by 2–4 weeks [69], to a maximum of 16 weeks (or potentially more with longer-acting anti-VEGF agents), however, there will be a higher risk of recurrence [67]. If a patient reaches stability at a particular treatment interval, this should be maintained over the long term if feasible. If the treatment interval is alternately being extended and reduced at each visit, the clinician can consider that the shorter of the two intervals is the more appropriate one for the patient and maintain this interval for a period of time before re-evaluating the patient’s treatment needs in due course. There is currently no evidence to support stopping anti-VEGF treatment in patients with stable disease, as disease activity will very likely recur, but the clinician may consider that continued long-term anti-VEGF therapy may not be appropriate or beneficial in patients with low vision who have fibrosis, extensive subretinal haemorrhage, subfoveal disruption of the external limiting membrane or the ellipsoid zone or central atrophy of the RPE.

## Discussion and conclusion

The aim of this consensus article is to consider the evidence and guidance currently available in the scientific literature on the role of fluid in the management of nAMD and provide recommendations as to how it might be integrated into everyday clinical practice based on the opinion of a panel of expert retinal specialists. Our understanding of the role of fluid in nAMD is still evolving and in some instances the observations reported in the scientific literature are conflicting and confusing. The treatment recommendations provided here are based on our best interpretation of the available data at this time. The resulting algorithm for the diagnosis and management of nAMD provides clear guidance on recommended diagnostic tools and what they can be used to identify, as well as a simple treatment pathway based on the T&E regimen. It aims to provide the best possible visual outcomes for patients whilst acknowledging the restrictions that are inevitably encountered in real-world clinical practice. Treatment decisions are made according to observations of fluid as a biomarker for disease activity in nAMD. This publication is not an exhaustive review of the T&E regimen, which varies in detail between publications, but provides a recommended version of the T&E regimen based on the combined clinical experience of the consensus panel, and guided by fluid.

The detection of fluid on OCT is generally used to imply the presence of a VEGF-related leak that the clinician could expect to respond to anti-VEGF therapy. However, in some cases, the fluid spaces seen on OCT may actually be structural changes such as outer retinal tubulation that are not responsive to anti-VEGF treatment [70]. Where this is suspected, strategies to confirm that fluid is VEGF-driven include monitoring patients shortly after treatment (e.g., 2 weeks after injection) to check for a short-lived treatment response, checking for leakage from the lesion using FA, and assessing whether the putative fluid worsens with an extended treatment interval.

There are a number of limitations associated with this review and consensus. The scientific evidence reviewed here is limited in that the literature search retrieved only one level 1 evidence trial (the FLUID study) that specifically aimed to evaluate the impact of fluid in the management of nAMD [19]. Even this study had limitations in terms of determining the effect of treating fluid versus leaving it untreated since patients were treated at every visit in both treatment arms. Interestingly, both the arms where SRF was more tolerated and the arm where it was treated more aggressively had relatively high and nearly identical injection frequencies (means of 15.8 and 17 injections over 2 years). The remainder of the evidence comes from a number of RCTs in which the effect of fluid on treatment outcomes was an observational, secondary or exploratory outcome or the subject of a post-hoc analysis, or in the form of lower level evidence from prospective but uncontrolled trials and retrospective chart reviews. For the purposes of this review, all publications that met the literature review inclusion criteria have been considered, regardless of the level of evidence.

The imaging technologies used in the studies included here have not remained constant over the time span of the literature review. OCT has evolved from time domain to spectral domain modalities, meaning that the observations reported by the earliest publications returned by the literature search are not directly comparable to the more recent publications. Several of the larger RCTs used these older imaging techniques, which may detract from the relevance of their findings to current practice. Other measured parameters may have also changed over time.

A final limitation of this work is that for the purposes of providing clear guidance that can be used on a day-to-day basis by the practising clinician, this review and consensus focusses only on the role of fluid in nAMD. In addition to fluid, a range of other morphological features visible on OCT such as external limiting membrane, ellipsoid zone and RPE disruption, and the presence of PED and hyperreflective material have been associated with poor visual acuity outcomes [23, 24, 27–29, 31, 35, 40, 42, 45, 49–52, 71–75]. A relationship has also been observed between visual acuity outcomes and the type of neovascularisation. At baseline, type 1 MNV is a predictor of better visual acuity following anti-VEGF treatment compared with other lesion types, and eyes with this type of lesion often have SRF [76]. These associated morphological findings are also important features with prognostic value which can co-exist with fluid. However, given the difficulties in visualising, identifying and consistently assessing some of these other features, we are of the opinion that fluid is the most practical and useful biomarker of VEGF upregulation and MNV activity in nAMD.

In conclusion, gaps exist in the scientific literature on the role of fluid in the management of patients with nAMD. The limitations described here highlight the real need for appropriately designed and executed studies to provide a standardised and detailed understanding of the appearance of different specific fluid manifestations and their consequences on clinical outcomes. However, it is quite clear that the primary treatment goal is to eliminate fluid as effectively as possible. Future research into this important area could provide valuable insights to direct optimal treatment to achieve this. In the meantime, following expert consideration of the evidence available, we recommend that patients with nAMD receive anti-VEGF therapy according to a T&E regimen with treatment intervals determined according to fluid-based disease activity parameters observed using OCT.
